# Treatment of Parkinson’s disease in the advanced stage

**DOI:** 10.1007/s00702-013-1008-y

**Published:** 2013-03-10

**Authors:** C. Ossig, H. Reichmann

**Affiliations:** Department of Neurology, Dresden University of Technology, Dresden, Germany

**Keywords:** Dyskinesia, Motor fluctuations, Advanced Parkinson's disease, Levodopa

## Abstract

Levodopa/Carbidopa, respectively, Levodopa/Benserazide is the most effective treatment for Parkinson’s disease and during the progress of the disease, patients will inevitably need to be treated with it. Nonetheless, after a certain time period most of the patients experience side effects. Mainly disturbing are motor and non-motor fluctuations and dyskinesia. Numerous options from changing the medication regimen, to continuos dopaminergic drug delivery via apomorphine or Duodopa pumps and stereotactical interventions are available. The physician’s responsibility is to choose the right therapeutic procedure for each timepoint of the patient’s disease. In this review, we provide an up to date overview of the available strategies.

## Introduction

As one of the main pillars in the therapy of Parkinson’s disease (PD), Levodopa/Carbidopa (LC), respectively, Levodopa/Benserazide (LB) is the mainstay in treatment, with the highest antiparkinsonian efficacy. LC/LB increases the survival rate and the quality of life and will have to be added to any treatment regimen in the longrun (Olanow et al. 2004). Side effects of LC/LB treatment comprise nausea, vomiting, orthostatic hypotension, cognitive impairment, psychosis and obsessive compulsive disorders (OCD). In the ELLDOPA study, Fahn (2005) was able to show the significant improvement of Parkinson’s patients, measured on the Unified Parkinson Disease Rating Scale (UPDRS), depending on the dose of LC/LB therapy. However, depending on the daily dose of LC/LB, the onset of motor fluctuations and dyskinesia is often in as little time as 5–6 months to 2 years (Stocchi and Rabey 2011; Fahn 2005). Due to this disease development and Levodopa-induced side effects (LID), therapeutic regimens have to be adjusted and different strategies have to be considered.

### Fluctuations and dyskinesia

The underlying pathophysiology of Levodopa-induced fluctuations and dyskinesia is unknown, but the overall opinion is that it might be due to a discontinuous stimulation of the striatal dopamine receptors as opposed to the continuous supply of dopamine in the healthy individual (Olanow et al. 2006). Especially younger patients, with a PD onset before the age of 50, with high intake of LC/LB for an increased duration of time are at risk for developing motor fluctuations and dyskinesia (Kostic et al. 1991). In addition, female sex seems to be a risk factor (Schrag and Quinn 2000). Motor fluctuations can present as “end-of-dose” or “wearing-off” phenomenon, sometimes with even unpredictable “off” episodes. In addition, a delayed “on” or no “on” response after medication intake may occur. Non-motor fluctuations can present not only as severe anxiety, restlessness and mood swings but also as physical symptoms like urinary disorder, hyperhidrosis, fatigue and sleep disorders (Barone et al. 2009; Antonini et al. 2008a). Dyskinesias may appear at the highest level of Levodopa effectiveness as “peak dose” dyskinesia, but may also present as biphasic dyskinesia, before and after LC/LB dose interval.

Once fluctuations and dyskinesia emerge, the pharmacodynamic response changes, resulting in a narrowing of the “therapeutic window” and a specific levodopa threshold is needed for a sufficient clinical response (Mouradian et al. 1989). Motor fluctuations can primarily be kept under control by increasing the daily dose of LC/LB, and hence, increase “on” time, by risking the occurrence of dyskinesia or applying more frequently smaller dosages of LC/LB, consequently reduce dyskinesia but venture to increase the “off” time. Shortening the intervals between the LC/LB intakes may, however, reduce the compliance of patients (Grosset et al. 2005). Before changing a current medical schedule, the patient should be reminded of the reduced bioavailability of LC/LB, when a protein-rich meal is consumed.

## Non-invasive therapeutical options

### Levodopa/Carbidopa or Levodopa/Benserazide and MAO-B Inhibitors

Dopamine concentrations can be increased by blockage of the monoamine oxidase-B (MAO-B) leading to a reduced metabolism of dopamine in the brain. Selegiline (SE) was the first MAO-B inhibitor approved by the FDA in 1996. Daily treatment with 10 mg SE leads to an improvement of 3 points on the total UPDRS and 1.7 points on the motor subscale of the UPDRS after 3 months (Parkinson Study Group 1993). Since the metabolites of SE, metamphetamine and amphetamine, are capable to inhibit the peripheral monoamine oxidase-A (MAO-A) and therefore hold a potential risk of dietary tyramine-provoked hypertensive crisis, high dosages of SE should be avoided. Adding 0.5–1 mg rasagiline (RA) to the LC/LB therapy results significantly in a reduced “off” time and an increase in “on” time as shown in the PRESTO (Parkinson Study Group 2005) and LARGO study (Stocchi and Rabey 2011; Rascol et al. 2005). The excellent side effect profile of RA has been confirmed in the ADAGIO trial, which showed no significant differences in adverse events between placebo treated patients and study participants receiving 1–2 mg of RA (Olanow et al. 2009). Data from preclinical studies have suggested that MAO-B inhibitors may have neuroprotective properties (Heikkila et al. 1984). Considering clinical studies, only the open-label extension of the TEMPO study demonstrated that early RA treatment provided a long-term clinical benefit, suggesting at least some rationale for a disease-modifying effect of RA (Hauser et al. 2009). However, the MAO-B inhibitors may reinforce the appearance of dyskinesia and lead to dopaminergic side effects.

### Levodopa/Carbidopa or Levodopa/Benserazide and COMT inhibitors

Levodopa is partly metabolised by the catechol-O-methyl transferase (COMT). Inhibitors of COMT increase the elimination half-life of Levodopa and boost the effect of each tablet by approximately 30 %. Two selective inhibitors are commonly used: Entacapone (a peripheral COMT inhibitor) and Tolcapone (a peripheral and central acting COMT inhibitor). Tolcapone possesses the ability of greater COMT inhibition (Deane et al. 2004) but since sporadic cases of hepatotoxicity have occurred, regular blood test is mandatory. Besides this distinctive feature, side effects resemble LC/LB. Adjunction of a COMT inhibitor to the LC/LB treatment leads to an increase in “on” time, a reduction in “off” time and an improvement of motor scores in the fluctuating PD patient (Parkinson Study group 1997). However, the adjunct of Entacapone to LC/LB failed to delay the time of onset or reduce the frequency of dyskinesia as shown in the STRIDE-PD study (Stocchi et al. 2010). In this double blind trial, every 3.5 h LC/LB or LC/LB plus entacapone (LE) was administered. Patients who received LE had an increased risk in developing dyskinesia compared to the control group. Dyskinesia under LE treatment were even more common when patients were younger than 65 years of age, of male sex, with a body weight >75 kg, with a disease duration less then 2 years and an additional treatment with a MAO-B inhibitor or dopamine agonist (Stocchi et al. 2010).

### Levodopa and dopamine agonists

The commonly oral used dopamine agonists (DA) are ropinirole, piribedil and pramipexole. Rotigotine is applied transdermally. DA is often applied as an initial treatment in PD due to their low potential to develop dyskinesia (Rascol et al. 2000). However, the majority of patients receiving DA will also need LC/LB after 2–5 years (Holloway et al. 2004). Being initially developed as an add-on therapy to LC/LB, several studies have shown a significant reduction of “off” time of about 1.1–1.5 h per day (Olanow et al. 1994; Pinter et al. 1999). Nowadays, mainly 24 h prolonged release formulations of DA are in use and have shown significant reduction of “off” time (Pahwa et al. 2007). Side effects resemble LC/LB treatment; however, increased daytime sleepiness and skin irritations in the transdermally applied form may occur. Recent reports have also linked DA therapy to pathologic gambling, eating disorders and hypersexuality (Stocchi 2005; Antonini and Cilia 2009). Interestingly, the use of ropinirole as initial therapy of early PD showed a reduced incidence of dyskinesia for up to 5 years, regardless of supplemental LC/LB therapy (Rascol et al. 2000). Recently, Watts et al. (2010) investigated that the addition of prolonged release ropinirole to LC/LB therapy delays the occurrence of dyskinesia. In addition, non-motor fluctuations can improve from add-on therapy of DA to LC/LB. The RECOVER study showed that adjunction of 24 h transdermal rotigotine treatment is associated with significant benefits in the management of early morning motor impairment, nocturnal sleep disturbances and non-motor daytime symptoms such as fatigue and mood changes (Trenkwalder et al. 2011).

### Therapeutic algorithm regarding non-invasive therapeutical options

**Fig. 1:**
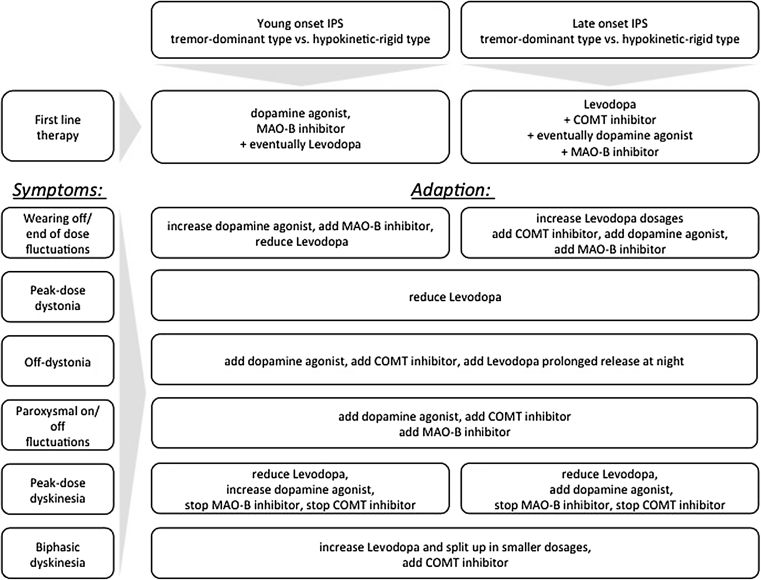
Therapeutic algorithm for orally applicated medication according to symptoms

Figure 1 offers an overview how treatment should be adjusted according to the occurrence of common subtypes of dyskinesia and motor fluctuations.

## Invasive therapeutical options

If symptoms cannot sufficiently be controlled by orally applicated medication, invasive therapeutical options must be considered. Continuous dopaminergic drug delivery (CDD) can be achieved by continuous subcutaneous apomorphine infusions (CAI) or duodenal infusions of Levodopa (Duodopa) via portable minipumps (Hilker et al. 2011). Constant dopaminergic stimulation of the receptors is associated with a reduced risk of motor fluctuations compared to treatment with intermittent dosages of standard oral LC/LB (Obeso et al. 1994). In healthy individuals, dopaminergic neurons in the substantia nigra fire at a constant frequency (Grace and Bunney 1984) and striatal dopamine levels are not subject to temporary fluctuations (Venton et al. 2003).

### Apomorphine

Apomorphine is a D1/D2 receptor DA with a short half-life of nearly 45 min and is, therefore, extremely effective considering time from injection to onset of clinical effects and mean duration of symptom relief (Neef and van Laar 1999). Apomorphine injections via pen administration are reasonable for a rapid relieve of a sudden “off” fluctuation during the daytime and nighttime and to conquer end-of-dose biphasic dyskinesia (Frankel et al. 1990). Continuous infusion can be enabled by an apomorphine pump. Via this modified insulin pump, a programmed infusion rate is dispensed in most cases using a 12–16 h regimen. If needed, the pump can administer apomorphine for 24 h and does not have to be discontinued overnight. This has resulted in a reduced nocturnal off-time (Reuter et al. 1999). Furthermore, a specifically defined bolus application can be given whenever needed. Treatment should be started under medical supervision and a premedication with domperidone should be utilised to reduce side effects (Bowron 2004). For analysis of circulating antibodies against erythrocytes, a Coombs test should be performed beforehand. One of the main side effects are skin reactions, located at the injection side resulting in a formation of small nodules (Deleu et al. 2004). Other side effects include increased daytime sleepiness, nausea, dizziness, renal impairment and orthostatic hypotension (Manson et al. 2001; Stocchi et al. 2001). Neuropsychiatric changes like hallucinations and psychosis are rare but can be observed with high dosages of apomorphine. CAI may provide a significant improvement of mood (Morgante et al. 2004).

### Duodopa

Duodopa is a combination of Levodopa (20 mg/ml) and Carbidopa (5 mg/ml) applied in form of a gel into the duodenum. Preceding the permanent therapy, a test application period of Duodopa via a nasoduodenal catheter system is generally used. If this is well tolerated by the patient and symptoms improve, a percutaneous, endoscopic gastrostomy (PEG) is performed and Duodopa is delivered via a portable pump and a duodenal catheter. If the retention period is 24 h, Duodopa is given as a monotherapy. If the Duodopa treatment is subject to a 16 h regimen, a prolonged release Levodopa tablet is often administered before bedtime. A number of studies have shown a significant reduction of time in “off” and a significant increase of time in “on”, as well as a reduction of dyskinesia (Antonini et al. 2008b; Eggert et al. 2008). In addition, an improvement of the non-motor symptoms including sleep and pain, gastrointestinal, urological and cognitive issues was stated (Honig et al. 2009). Adverse events are mainly due to technical reasons like dislocation, obstruction and breakage of the duodenal catheter. In very rare cases, PEG related side effects are monitored. These include peritonitis and local stoma inflammation.

### Deep brain stimulation

The use of continuous high frequency “deep brain” stimulation (DBS) was established in the early 90s and is widely used with great benefit (Benabid et al. 1994). The implanted electrodes can be adjusted by changing the voltage, the pulse width and the frequency to improve symptoms and/or reduce potential side effects. In PD patients, the stimulation of either the subthalamic nuclei (STN) or the internal global pallidum (GPi) is mainly performed. The duration of benefit following GPi DBS is, however, variable. After STN DBS, an improvement in all main cardinal features of PD as well as a reduction of the mean severity and duration of dyskinesia has been documented in several studies (Limousin et al. 1998; Krack et al. 2003; Weaver et al. 2012). The best predictor for a favourable result is a good Levodopa response (Charles et al. 2002). Ideal candidates for STN DBS are patients below the age of 70 years, with motor fluctuations and dyskinesia or tremor and without cognitive or behavioural deficits. Side effects of the DBS include intracranial haemorrhage and infarction. Postoperational complications involve confusion, pneumonia, infection and in older patients pulmonary embolism (Voges et al. 2007). Lead breakage, extension wire failure, impulse generator malfunctions—generally referred to as hardware-related complications—typically appear within the first 3 months after operation (Baizabal Carvallo et al. 2012; Lyons et al. 2004). Stimulation-induced side effects include dysarthria, hypophonia, dizziness, eyelid opening apraxia and oculomotor deficits (Deuschl et al. 2006). Neuropsychiatric changes like post surgery depression and especially a 13-fold increased risk for suicide within the first year after STN DBS have been the cause for concern (Voon et al. 2008). A recent study did not find a difference in functional health measured by time spent in “on” and “off”, behavioural side effects, cognition and mood in patients who received GPi DBS or STN DBS (Odekerken et al. 2013).

### Conclusion regarding invasive therapeutical options

**Fig. 2:**
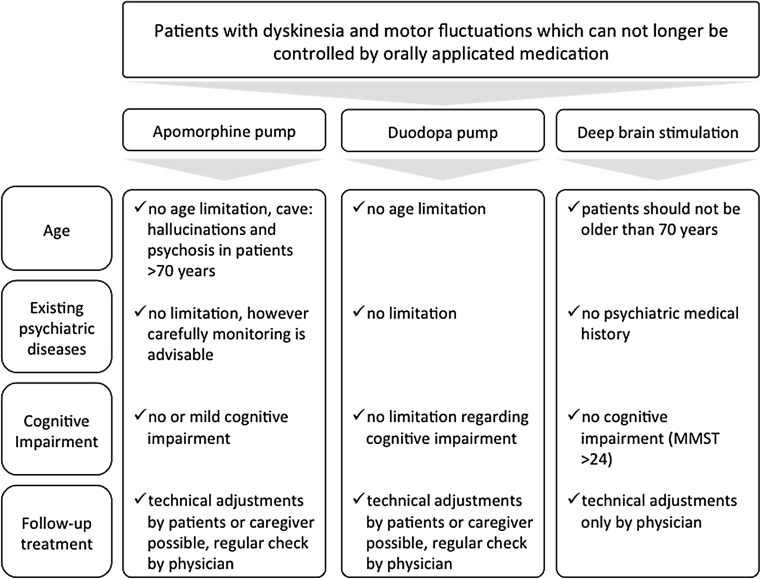
Invasive therapeutical options for advanced Parkinson’s disease treatment

The indication for any invasive therapeutical option—continuous infusion of apomorphine or Duodopa via pumps or DBS via stereotactical operation—should be carefully placed (compare Fig. 2). As apomorphine pumps are small and of lower weight compared to Duodopa pumps, it need less technical requirements and are generally easy to apply and completely reversible. CAI is often used as an interim solution before DBS. The side effects can be generally controlled well; however, a continuation of the orally administered medication is almost always necessary. Until today comparative studies with CAI and Duodopa have not been accomplished. However, the effect of Duodopa on “off” time is distinct. DBS shows great benefit in controlling PD symptomatology and offers more independence for patients than treatment with pumps; however, the indication for undergoing stereotactical operation must be strictly adhered to receive a satisfying outcome. For a good clinical outcome with any invasive therapeutical option, a high patient motivation is needed, a necessary detailed patient education, regular follow-up visits in a specialised setting and a stable support of the patient by his environment.
